# A millifluidic bioreactor allows the long term culture of primary lymphocytes or CD34^+^ hematopoietic cells while allowing the detection of tumorigenic expansion

**DOI:** 10.3389/fbioe.2024.1388312

**Published:** 2024-10-02

**Authors:** Paolo Ritter, Stefania Oliveto, Chiara Cordiglieri, Alessandra Fasciani, Christian Andrea Di Buduo, Lucrezia della Volpe, Alberto Bocconi, Claudio Conci, Carolina Paula Miguel, Raffaella Di Micco, Alessandra Balduini, Manuela Teresa Raimondi, Stefano Biffo

**Affiliations:** ^1^ National Institute of Molecular Genetics, Fondazione Romeo ed Enrica Invernizzi, INGM, Milan, Italy; ^2^ Department of Chemistry, Materials and Chemical Engineering “Giulio Natta”, Politecnico di Milano, Milan, Italy; ^3^ Department of Biosciences, University of Milan, Milan, Italy; ^4^ Department of Molecular Medicine, University of Pavia, Pavia, Italy; ^5^ San Raffaele Telethon Institute for Gene Therapy, IRCCS San Raffaele Scientific Institute, Milan, Italy; ^6^ University School for Advanced Studies IUSS, Pavia, Italy

**Keywords:** CD4^+^, CD34^+^, gene therapy, fluidics, bone marrow

## Abstract

Long-term culture of primary lymphocytes and hematopoietic stem and progenitor cells (HSPCs) is pivotal to their expansion and study. Furthermore, genetic engineering of the above-mentioned primary human cells has several safety needs, including the requirement of efficient *in vitro* assays for unwanted tumorigenic events. In this work, we tested and optimized the Miniaturized Optically Accessible Bioreactor (MOAB) platform. The MOAB consists of a millifluidic cell culture device with three optically-accessible culture chambers. Inside the MOAB, we inserted a silk-based framework that resembles some properties of the bone marrow environment and cultivated in this device either CD4^+^ T lymphocytes isolated from healthy donor buffy coat or cord blood-derived hematopoietic CD34^+^ cells. A fraction of these cells is viable for up to 3 months. Next, we tested the capability of the MOAB to detect tumorigenic events. Serial dilutions of engineered fluorescent tumor cells were mixed with either CD4^+^ or CD34^+^ primary cells, and their growth was followed. By this approach, we successfully detected as little as 100 tumorigenic cells mixed with 100,000 primary cells. We found that non-tumorigenic primary cells colonized the silk environment, whereas tumor cells, after an adaptation phase, expanded and entered the circulation. We conclude that the millifluidic platform allows the detection of rare tumorigenic events in the long-term culture of human cells.

## 1 Introduction

*In vitro* cell culture on two-dimensional (2D) glass or plastic substrates has been widely used and has significantly influenced cell biology studies. Despite their simplicity, 2D cultures have several limitations and fail to model the complex physiological three-dimensional (3D) environment and to reproduce *in vivo* cell behavior (Antoni et al., 2015; Ravi et al., 2015; Bissell, 2017; Kapałczyńska et al., 2018; Fontoura et al., 2020). Furthermore, the absence of a continuous medium flow does not provide an efficient nutrient supply and waste removal. To address the shortcomings of 2D cultures, several 3D systems have been developed. Overall, 3D systems aim to replicate the natural environment of cells, making them more representative of actual cellular environments. Therefore, they are utilized in diverse areas such as tissue engineering, drug discovery, and cancer research (Fang and Eglen, 2017; Oliveto et al., 2024). However, most 3D culture systems currently lack the capability to supply cultures with flow, which is essential for the analysis of cells that grow under laminar flow conditions. Different types of bioreactors aiming at solving this issue have been proposed (Cabrita et al., 2003; Born et al., 2021; Sommerkamp et al., 2021). In this context, a Miniaturized Optically Accessible Bioreactor (MOAB) was used to recreate the interstitial perfusion by using 3D cell constructs (Laganà and Raimondi, 2012). This miniaturized, optically accessible perfusion bioreactor allows the culture of 3D organoids, up to a few millimeters in size, under continuous perfusion of the culture medium (Raimondi et al., 2015; Izzo et al., 2019; Steimberg et al., 2020; Ene-Iordache et al., 2021). This setup may allow, for instance, the construction of a bone marrow-like structure continuously perfused, as *in vivo* (Marturano-Kruik et al., 2018).

Bone marrow is a semi-solid tissue found within the spongy portions of bones. It is the primary site of new blood cell production, also known as hematopoiesis. The bone marrow is composed of hematopoietic cells, marrow adipose tissue, and supportive stromal cells. Hematopoiesis is initiated by hematopoietic stem cells (HSCs). These cells possess the unique ability to give rise to all the different types of mature blood cells and tissues (Comazzetto et al., 2021). In addition, *in vivo,* differentiated cells leave the bone marrow and enter the blood circuit. Standard and 3D cultures of hematopoietic cells performed in a static phase, lead to restricted cell expansion (Tripathi et al., 2020). Several attempts to reconstruct the bone marrow environment are underway, including vascularized ones (Glaser et al., 2022). All systems face the challenge of long-term culture and cell expansion. A programmable, bioengineered, three-dimensional silk-based bone marrow niche tissue system has been described (Di Buduo et al., 2015; Di Buduo et al., 2017; Di Buduo et al., 2021). These systems mimic the physiology of the human bone marrow environment and allow the differentiation of platelets (Di Buduo et al., 2015). We reasoned that the connection of this system to a circulating medium may allow the clonal expansion of selective cell populations.

The transplant of HSPCs is an established standard of care intervention for hematologic malignancies, some neoplastic disorders, and severe immunologic deficiencies. In well-established allogeneic settings, bone marrow-derived HSPCs are retrieved from a donor and transplanted into a recipient. This type of intervention requires several meeting criteria, in order to reduce graft versus host disease (GvHD) (Ferrara et al., 2009). A breakthrough in hematopoietic stem cell transplant is possible by autologous stem cell transplant. In this last case, HSPCs are first retrieved from the patient, then corrected by gene therapy *in vitro*, and finally grafted into the same patient (Ferrari et al., 2021). However, insertional mutagenesis, combined with acquired somatic mutation events, was found to cause leukemogenesis following gene therapy (Hacein-Bey-Abina et al., 2003; Howe et al., 2008). Indeed, gene editing, including techniques like CRISPR, carries the risk of tumorigenic events. These risks primarily stem from off-target effects, where unintended alterations occur in the genome. Such alterations can influence intercellular communications and interactions, potentially leading to the development of tumors (Kaiser, 2020). Unfortunately, pro-tumorigenic alterations can be subtle and not detectable after editing, thus leading to disease only after the grafting of HSPCs *in vivo*. For these reasons, gene therapy associated with bone marrow transplants is reserved for highly compromised patients. In order to make the intervention safer and accessible to more patients, efficient systems for detecting unwanted tumorigenic events are needed. The conventional approach for tumorigenicity is based on *in vivo* tumorigenicity assays in which cells are implanted at an ectopic site in immunodeficient mice and monitored for the formation of tumor masses (Sato et al., 2019). However, these methods are not highly sensitive in the detection of pre-tumorigenic events that could be counter-selected in the highly selective *in vivo* environment. Indeed, oncogenic mutations may remain silent for years and cause disease in individuals who endure another genomic hit, or be selected in disease settings in which clonal expansion of the edited cells is triggered by a selective advantage conferred by gene correction. In addition to immunodeficient mice, other methods for predicting tumorigenicity are growth in soft agar (Ke et al., 2004) or sequencing analysis (Salk and Kennedy, 2020) which suffer, respectively, from very high stringency and analytical complexity and sensitivity. Thus, the need for an easy experimental model that favors and allows the detection of tumorigenic cells clonal expansion is urgent. To bridge this gap, we propose utilizing a miniaturized bioreactor to test the eventual expansion of cells carrying unwanted protumorigenic mutations after gene-editing. This model helps in predicting and detecting potential malignant phenotypes, providing a controlled environment for long-term observation of edited cell populations.

In this study, we implemented a dynamic *in vitro* model that allows for long-term cultivation and is suitable for different primary human cell cultures. This model consists of a bioreactor, the MOAB, that hosts a silk scaffold seeded with primary human cells while allowing medium perfusion and optical accessibility. The scaffold was employed for the biomimicry of the extracellular matrix component of the bone marrow and, thus, provides the 3D environment for cells to grow in. Besides, the medium perfusion yielded by the bioreactor mimics the blood flow in supporting the oxygen and nutrient delivery and facilitating, at the same time, the removal of waste products, allowing for long-term studies. In particular, we have validated the platform for long-term culture of human primary cells, such as CD4^+^ T lymphocytes and CD34^+^ hematopoietic stem cells. Regarding the tumor cells, we used a peripheral blood acute myeloid leukemia line (PLB-985). Importantly, we show that the platform enables the detection and expansion of tumor cells in both lymphoid and hematopoietic niches. Thus, it fulfills its potential as a validated test for identifying unwanted tumorigenic events during the gene correction process.

## 2 Materials and methods

### 2.1 System setup

In this section, we have explored the complexities of the perfusion circuit, a critical component of our cell culture system. This circuit, composed of three main elements, plays a pivotal role in maintaining optimal conditions for cell growth and development. A peristaltic pump draws culture medium from the gravitational reservoir, actively perfuses the Miniaturized Optically Accessible Bioreactor (MOAB) (MOAB Srl, Italy), and finally, returns the exhausted medium to a gravitational reservoir. Here, due to the presence of a channel equipped with a filter, the oxygen concentration in the medium is restored to its equilibrium value. The pump is fitted with 20 cm tubing of a specific internal diameter (ID = 1 mm) that ensures controlled flow rates. This tubing is connected via Luer lock connectors and 30 cm oxygenator tubing on one side of the pump to the reservoir outlet and on the other side to the MOAB inlet. Another set of 30 cm oxygenator tubing, fitted with Luer lock connectors, is used to connect the MOAB outlet to the gravitational reservoir inlet. The pump operates at a rate of 0.3 rpm, corresponding to 18 μL/min (using 1 mm × 1 mm ID × wall thickness silicone tubing) during cell culture.

### 2.2 Dynamic cell culture conditions

Cell cultures were maintained in a humidified environment at 37°C with 5% CO_2_. Cell mixtures were prepared for seeding according to the desired final ratios. The mixed cells were counted and then suspended in a fresh medium. A maximum of 100,000 cells were resuspended in a final volume of 30 μL of complete StemSpan™ SFEM (Stemcell Technologies, Canada). These cells were directly seeded onto the fibronectin-functionalized silk fibroin scaffolds housed in the MOAB lid. Silk scaffolds have been fabricated according to previously described methods (Di Buduo et al., 2018). After seeding, the lid is incubated in a Petri-in-Petri approach having up to three 60 mm Petri dish bottoms, each containing one MOAB lid, contained in a 150 mm Petri dish filled with PBS 1X (EuroClone, Italy) to ensure high humidity and low evaporation of the medium from the scaffolds. After 72 h of incubation, during which the scaffold is flipped every 24 h, the lids are coupled to the bioreactor thus originating the 9 uL 3 mm × 6 mm × 0.5 mm culture chamber, and the system is then perfused.

### 2.3 Circuit priming

Circuit priming is the earliest operation performed before coupling the lids with the MOAB which prevents the scaffold from drying due to prolonged airflow. This process involves refilling the reservoir with 7 mL of complete StemSpan™ SFEM (Stemcell Technologies, Canada), activating the pump, and increasing its speed to 20 rpm until the medium reaches the interface of the bioreactor chamber. Once this is achieved, the pump is stopped, the speed is reduced to 0.3 rpm, and the MOAB lids are coupled with the main body of the bioreactor. The system is then positioned adjacent to the incubator, and the pump can be activated.

### 2.4 Medium change

Every 5 days of continuous perfusion, a medium change is performed. To carry out this task, the pump is stopped, and tubings are clamped to prevent unwanted flow when handling the circuit. The system is then moved under a sterility hood. The circuit is opened, and the exhausted medium is withdrawn from the reservoir outlet using a sterile syringe. Fresh medium is then added. After all the connectors have been tightened, the system is placed back in the incubator and fastened to the pump, which is then activated. At this point, the clamps on the tubings are removed.

### 2.5 Cells and culture conditions

Cell lines were maintained following standard tissue culture protocols. Culturing of cells was performed in a humidified environment at 37°C with 5% CO_2_. All culture media were supplemented with 1% penicillin–streptomycin (EuroClone, Italy). PLB-985 (HL-60 subclone) leukemic cells were obtained from ATCC (United States) and grown in RPMI 1640 medium (EuroClone, Italy) supplemented with 10% FBS (EuroClone, Italy), 2 mM L-glutamine (EuroClone, Italy). CD4^+^ T lymphocytes were cultivated in RPMI 1640 medium (EuroClone, Italy), supplemented with 10% FBS (EuroClone, Italy), MEM Non-essential Amino Acids (Euroclone, Italy), Sodium pyruvate (1 mM, Euroclone, Italy), L-glutamine (EuroClone, Italy). PLB-985 educated cells, CD4^+^ T lymphocytes, and CD34^+^ HSPCs were cultivated in StemSpan™ SFEM (STEMCELL Technologies, Canada) supplemented with Human Stem cell factor (SCF) (100 ng/mL, PeproTech, Inc., United States), Human Flt3 ligand (Flt3) (100 ng/mL, PeproTech, Inc., United States.), Human Thrombopoietin (TPO) (20 ng/mL; PeproTech, Inc., United States), Human Interleukin 6 (IL-6) (20 ng/mL; PeproTech, Inc., United States) and Stem Regenin 1 (SR1) (1 μM; BioVision Inc., United States). Long-term cultured CD34^+^ cells will be named as HCs (Hematopoietic Cells) to acknowledge that they might lose CD34 expression over time.

### 2.6 Cell viability and cell proliferation analysis

The viability of cells was evaluated using a 0.4% Trypan blue stain (Gibco) exclusion test, as reported in (Strober, 2015). Cell proliferation was assessed by flow cytometry analysis using Cell Trace™ CFSE (Thermo Fisher Scientific, Invitrogen, Italy). In detail, 10^6^ cells were suspended in 1 mL of PBS 1X (Euroclone, Italy), and 1 µL of Cell Trace™ CFSE (Thermo Fisher Scientific, Invitrogen, Italy) was added, followed by incubation for 15 min at 37°C. After the incubation, 5 mL of culture medium was added, and the samples were left for 5 min at room temperature. The cells were then centrifuged at 300 rcf for 5 min, resuspended in MACS buffer (Miltenyi Biotec, Italy), and analyzed by flow cytometry (Oliveto et al., 2018).

### 2.7 Apoptosis assay

Cell apoptosis was analyzed by flow cytometry using BD Pharmingen™ APC Annexin V (Becton Dickinson Italia, Italy) and BD Pharmingen™ Propidium Iodide Staining Solution (Becton Dickinson Italia, Italy) (Oliveto et al., 2018; De Ponte Conti et al., 2021). As a positive control, we used cells treated with 200 nM Staurosporine (Merck Millipore, Germany) for 24 h. In detail, all samples analyzed were suspended in Annexin-Binding Buffer to obtain a dilution of 10^6^ cells/mL. Annexin V and Propidium Iodide were added to the cell suspension and incubated for 15 min at room temperature in the dark, following the manufacturer’s instructions.

### 2.8 CD4^+^ T lymphocytes isolation

Peripheral blood mononuclear cells (PBMCs) were isolated from buffy coats of healthy donors in compliance with approved protocol IMMUNOM (Fondazione IRCCS Ca’ Granda Ospedale Maggiore Policlinico, Milan) by Ficoll-Paque^®^ PLUS (VWR, United States) density gradient centrifugation. CD4^+^ T lymphocytes were enriched from PBMCs through magnetic cell separation using a CD4^+^ isolation kit (Miltenyi Biotec, Italy) and AutoMACS^®^ columns (Miltenyi Biotec, Italy) (Ricciardi et al., 2018). CD4^+^ T cells were purified by flow cytometry sorting using CD4 Antibody, anti-human, VioGreen™ (Miltenyi Biotec, Italy) at a dilution of 1 μL/10^6^ cells. They were finally labeled with CellTrace FarRed (Thermo Fisher Scientific, Invitrogen, Italy) according to the manufacturer’s instructions.

### 2.9 CD34^+^ HSPCs isolation

CD34^+^ HSPCs were either freshly purified from human healthy donor cord blood (Murga et al., 2004) after obtaining informed consent according to the TIGET09 protocol (approved by Ospedale San Raffaele Bioethical Committee) or purchased from Lonza, Switzerland. CD34^+^ HSPCs were isolated through magnetic cell separation using the CD34 MicroBead Kit UltraPure (Miltenyi Biotec, Italy), MS Columns (Miltenyi Biotec, Italy), and a MACS Separator (Miltenyi Biotec, Italy). CD34^+^ HSPCs were magnetically labeled with CD34 MicroBeads, and then the cell suspension was loaded onto the MACS Column. Magnetically labeled CD34^+^ cells are retained within the column and were eluted as the positively selected cell fraction, as reported in the datasheet.

### 2.10 Lentiviral transduction

PLB-985 leukemic cells (Tucker et al., 1987) were stably transduced with lentiviral vectors (Gandin et al., 2008) containing the GFP and the mCherry gene, separately. The transduction process was facilitated by the spinfection method. In detail, cells and viral particles were centrifuged at 400 rcf for 60 min at 37°C. After 3 days, the transduced cells were sorted using a FACSaria III cell sorter (BD Biosciences, United States). CD34^+^ HSPCs were seeded at 10^6^ cells/mL, treated with 8 µM Cyclosporin H (Sigma Aldrich, Germany), and then transduced with a PGK. GFP lentiviral vector with a multiplicity of infection (MOI) of 30. The day after the transduction, the medium was changed. Cells were then sorted gating for GFP^+^ events as shown in Supplementary Figure S1, and after 7 days of culture, they were seeded into a silk scaffold.

### 2.11 Immunofluorescence

Anti-CD3 primary antibody (Biolegend, United States) was used as a membrane marker to detect intact T cells. Briefly, after 3 months of dynamic culture, the cells populating the scaffold were fixed in paraformaldehyde (Sigma Aldrich, Germany), and after blocking in PBS 1X + BSA 2% (Merck Millipore, Germany), they were incubated with primary antibody, washed and the reaction revealed with fluorescent conjugated secondary antibody (Thermo Fisher Scientific, Invitrogen, Italy).

For immunofluorescence imaging of fibronectin-functionalized silk BM scaffold, samples were fixed in 4% PFA for 20 min and then blocked with 5% bovine serum albumin (BSA, Sigma) for 30 min at RT. Samples were probed with anti-fibronectin (1:250) (Merck Life Sciences), overnight and then immersed in Alexa Fluor secondary antibody (1:200) for 2 h. For silk BM scaffold imaging, we took advantage of silk auto-fluorescence in UV light. Samples were imaged by a TCS SP8 confocal laser scanning microscope (Leica). 3D reconstruction and image processing were performed using Leica-licensed software.

### 2.12 Imaging analysis

**FIGURE 1:**
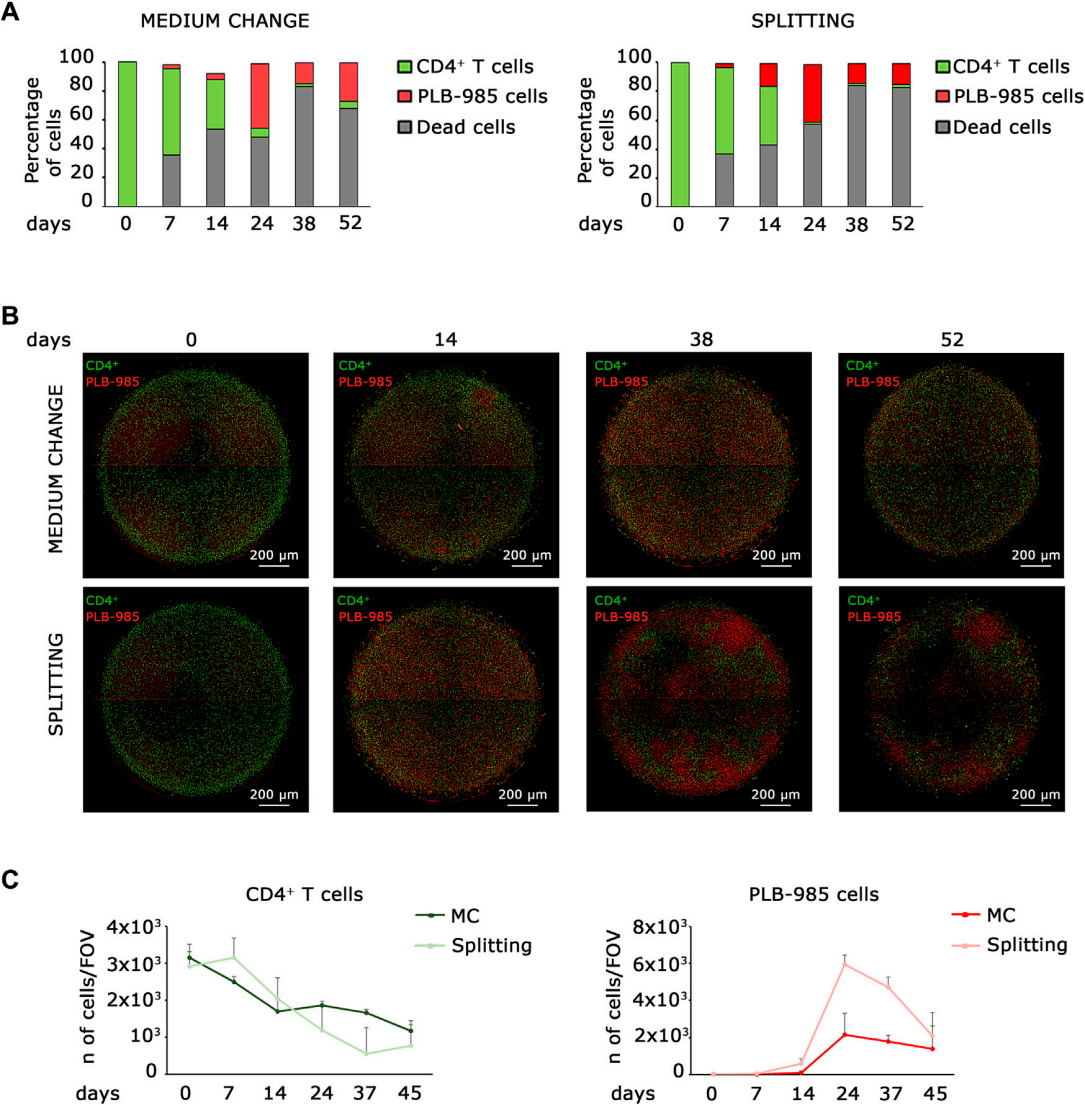
Co-culture static models identify early stages of tumorigenic events, but fail at long time points. **(A)** Flow cytometry analysis of leukemic cells (PLB-985) and lymphocytes CD4^+^ T cells in co-culture reveals that while CD4^+^ T cells exhibit a decline over time, the count of leukemic cells increases under both medium change (left) and splitting conditions (right). However, the number of dead cells increases over time. **(B)** Representative images of the evolution over time of the two co-culturing conditions: medium change (top row) and splitting (bottom row). Leukemic cells (red Look-Up Table, LUT) show an early expansion already after 14 days, while CD4^+^ T cells (green LUT) decrease over time. However, after 52 days both the cell types show a significative diminishing. Images are mosaics of 4 tiled field of views (FOVs, ×4 objective). Scale bars are shown. **(C)** Plot of the average cell count from 5 distinct FOVs, obtained from the imaging analysis of leukemic cells (PLB-985) and CD4^+^ T cells in co-culture. Both the medium change and splitting conditions confirm a decrease in the number of CD4^+^ T cells (left) and an increase in leukemic cells (right) over an extended period. However, the system is inherently limited by the progressive loss of viability at longer time points. The specific time points are indicated.

Thanks to its optical accessibility, the MOAB was inspected by high-resolution imaging using a Video-Confocal-SuperResolution Microscope composed of a widefield inverted microscope (TiE model; Nikon Instruments, Europe) equipped with a spinning disk scanning head for confocal and structure illumination microscopy (model X-Light2-VCS; CREST Optics, Italy) connected to double camera detection with Andor EM-CCD DU888 and Andor Zyla sCMOS (Andor Technologies, Oxford Instruments, United Kingdom) and double excitation device with 4-solid state diode lasers (LDI) and 16-channel LEDs (CoolLed), for fast and high-resolved microscopy. The whole chamber was inspected by acquiring via ×4 objective (NA 0.45, Nikon Instruments, Japan) mosaics of tiles on the xy plane FOVs, and 5-micron z-step sectioning was performed to ensure that the whole dispersion of cells in the scaffold was captured. Representative areas of interest were further imaged using a ×10 objective (NA 0.75, Nikon Instruments, Japan) Afterward, FOV tiles in each z-plan were stitched together via NIS-elements (v5.30; Nikon Instruments-Lim l.t.d., Japan) and the maximum intensity z-projection was obtained via ImageJ 2.9.0/1.53 t (Schindelin et al., 2012). The image acquisition was performed every 7 days for the experiments shown in Figure 1, whereas for the experiments shown in Figures 4, 6, acquisitions were taken every month. In both cases, image analysis was performed using NIS-Elements software v.5.30 (Nikon Instruments, Japan) to obtain quantifications of the number of cells present within the MOAB scaffold via *ad hoc* designed pipelines of segmentation and object classification on the basis of fluorescence intensities. For better object detection, pre-processing steps of contrast-enhancement and background removal were employed, followed by proper thresholding for specific signal binarization and binary post-processing to perfectionate object definition and classification according to morphology and intensity, as shown in Supplementary Figures S2, S3. Counts of cellular objects were then obtained and further elaborated and graphically plotted (Miluzio et al., 2023).

### 2.13 Statistical analysis

Each experiment was repeated at least three times as independent biological replicates; means and SDs were calculated.

## 3 Results

### 3.1 Dynamic systems have a pivotal role in comprehending long-term cellular processes and tumorigenicity

Established tumorigenicity assays using *in vitro* cell culture are soft agar colony-forming assay, growth in low attachment assay, and 3D cell culture (Kronemberger et al., 2021), but none of them is readily applicable to hematopoietic and lymphoid cells. Here, we aim to describe a new assay for detecting tumorigenicity in *in vitro* hematopoietic and lymphoid niche-like settings. Ideally, a simple system is based on seeding a complex mixture of primary cells, allowing enough time for the emergence of a tumorigenic event, and scoring for the clonal expansion of a tumorigenic cell. The soft agar forming assay meets these requisites, but is very stringent and misses subtle alterations that predispose to cancer. First, we analyzed whether a static system could fill this gap. Thus, we analyzed the behavior of leukemic cells seeded at low density into a lymphoid niche containing normal cells, in a classical static model. We seeded 100,000 primary CD4^+^ T lymphocytes in a well of a 96-well plate for coculture with 100 leukemic cells and monitored them for approximately 50 days. We analyzed two different culture conditions for all established time points, which we defined as “splitting” when we kept cells at the desired density by progressive dilution and “medium change” when we did not dilute the cells over time. In order to distinguish CD4^+^ T lymphocytes from leukemic cells, we stained them with a green and red fluorescent marker, respectively. We analyzed cocultured cells by FACS (Figure 1A) and microscopy (Figures 1B, C). As shown in Figure 1A, the number of primary lymphocytes decreases over time; on the contrary, the number of leukemic cells increases, both in medium change and splitting conditions, thus indicating the progressive expansion of the tumor population. Microscopic analysis confirmed that lymphocytes and leukemic cells exhibit opposite trends; as the number of leukemic cells increases, the number of lymphocytes decreases (Figures 1B, C). However, both systems failed with long-term cultures. First, dead cells increase over time (Figure 1A); second, the number of cells in the system shows a marked decrease in long-term culture under both the medium change and splitting conditions, as shown in Figure 1C. These data suggest that static models cannot be employed for scoring a tumorigenic population that slowly takes over in the culture since they a) may become rapidly saturated and/or b) require dilution that may lead to loss of cells and/or an increase in the needed number of cultures.

### 3.2 The integration of dynamic culture principles with the MOAB generates a system that facilitates the maintenance of long-term cell cultures

**FIGURE 2:**
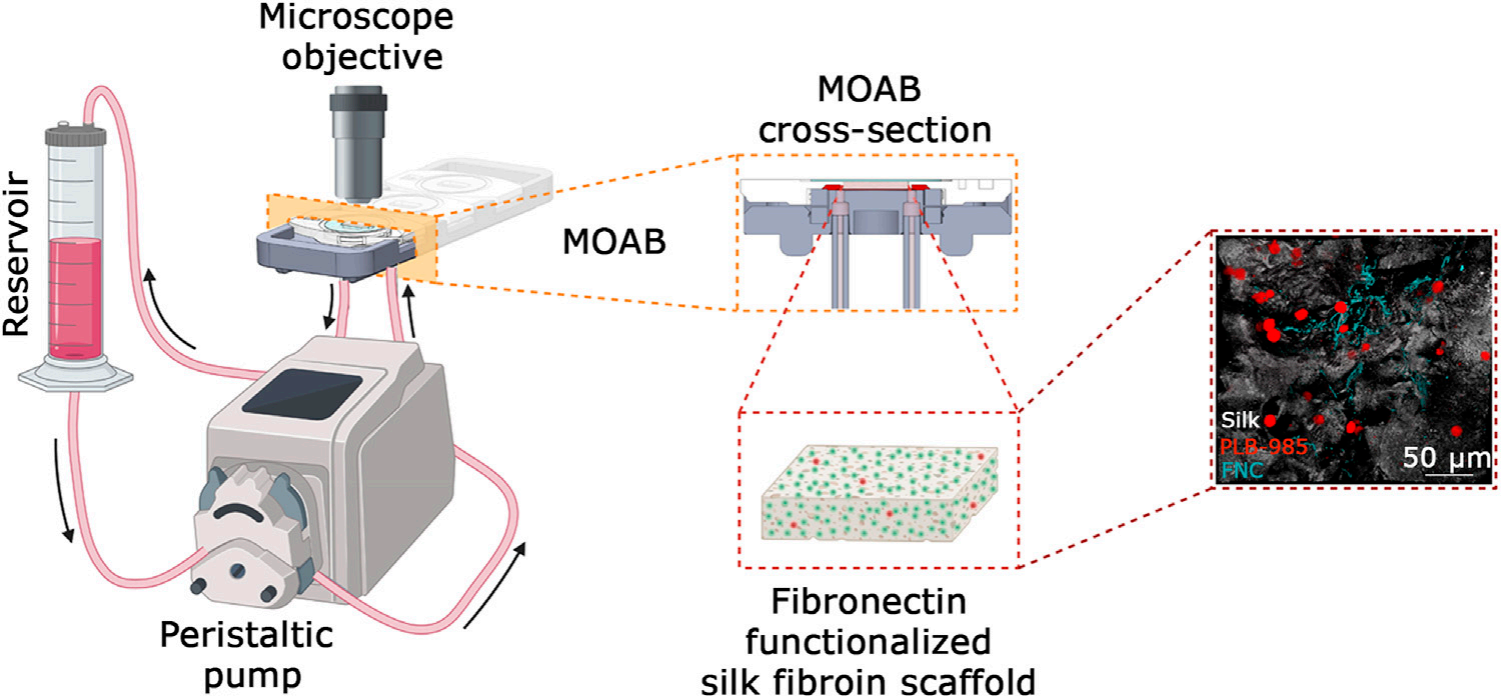
System setup. Cartoon of the system setup. It is composed of a reservoir for holding the culture medium withdrawn from a peristaltic pump and perfused through the MOAB, where the scaffold with seeded cells is housed. During the culture, cells are analyzed via imaging techniques thanks to the optical accessibility of the bioreactor. A detail of the MOAB section is evidenced. On the right is reported a representative fluorescence imaging of the model of bone marrow composed of leukemic cells (red LUT), fibronectin (cyan LUT) and silk fibroin (white LUT). In the cartoon it is represented one channel actively perfusing only one chamber of the bioreactor. The system is scalable up to three channels. Scale bar is shown. Created in BioRender.com.

To solve the issue of long-term culture, we opted for the adaption of an existing bioreactor, the MOAB. The scheme is shown in Figure 2 and detailed in Section 2.1 of Materials and Methods. We realized a long-term perfusion circuit availing of the use of three main components: the peristaltic pump, the MOAB, and the gravitational reservoir. In detail, the peristaltic pump withdraws the culture medium from the gravitational reservoir, actively perfuses the MOABs, and lastly, pushes the exhausted medium back to the gravitational reservoir. Thanks to the presence of a channel equipped with a filter, oxygen concentration in the medium is restored to its equilibrium value. The humidified incubator concentration is 18.6%, corresponding to dissolved oxygen concentrations lower than 200 μM (Place et al., 2017). Furthermore, the reservoir’s design facilitates cell sedimentation at its bottom, strategically positioned for withdrawal and subsequent perfusion through the bioreactor, completing a sophisticated and effective setup for dynamic cell culture investigations. Each MOAB contains three chambers that can be either connected in series or parallel. We connected them in series. Inside the chamber, we inserted a fibronectin functionalized scaffold, previously developed as an *ex vivo* 3-dimensional (3D) tissue model of the bone marrow niche environment (Di Buduo et al., 2015; Di Buduo et al., 2017; Di Buduo et al., 2021).

### 3.3 Construction of positive control and setting of a tumoral cell detection index (I_d_)

**FIGURE 3:**
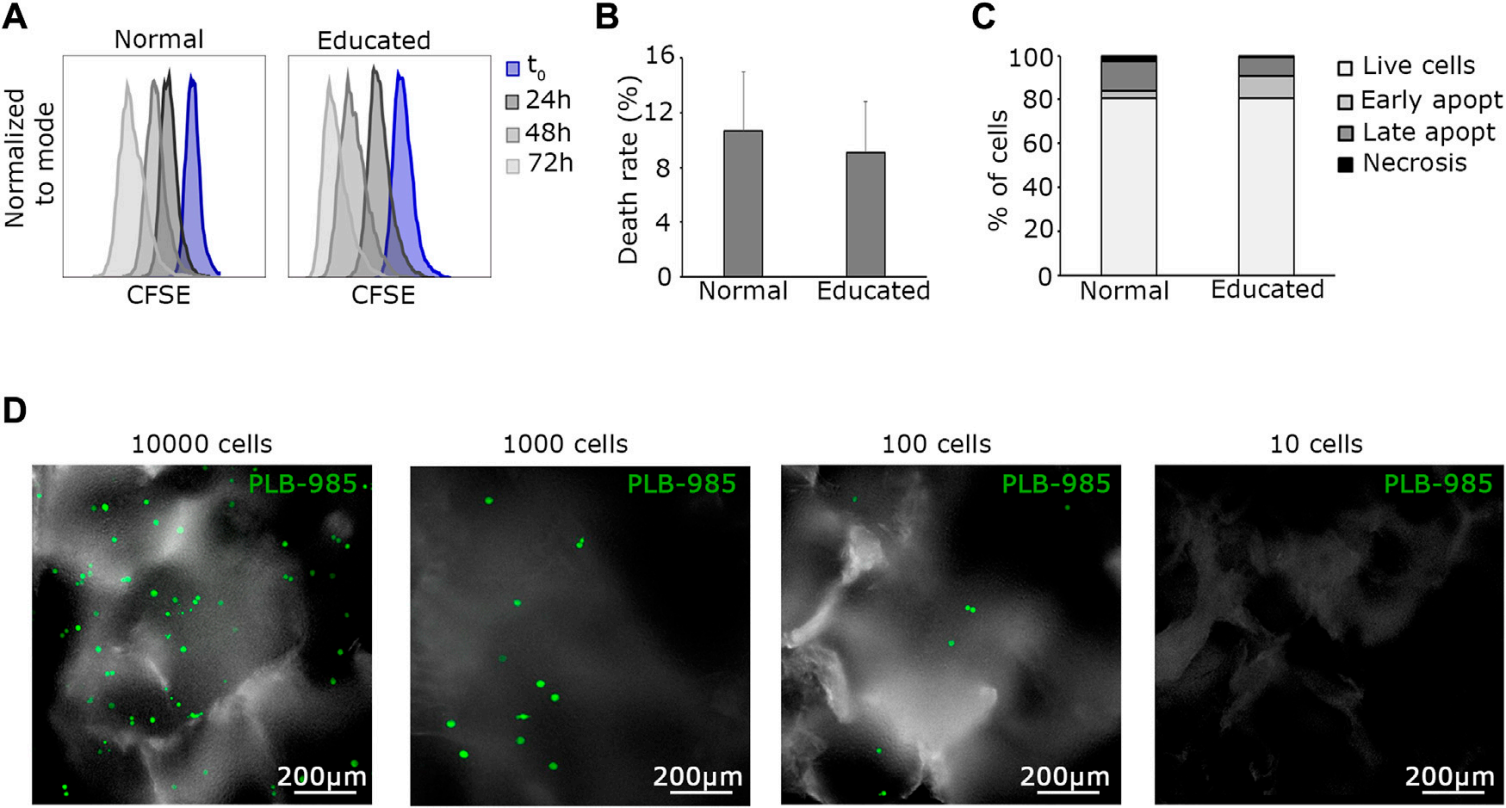
The tumoral detection index (I_d_) is as low as 100 cells. **(A)** Representative flow cytometry plots show the proliferation of normal and educated leukemic cells. There is no significant difference in the proliferation rate in the two conditions. **(B)** Death rates of normal and educated leukemic cells are similar, evidencing no difference among the two conditions. **(C)** FACS analysis of apoptotic events confirms that educated PLB-985 leukemic cells are fully viable. **(D)** Visualization of representative PLB-985-GFP^+^ cells seeded in the system at different dilutions. Images are live acquisitions at the final time point of 15 days. One hundred cells is the limit number for easy detection of this setup. In the 10 cells condition (far right) no cells are recognizable in the system. Scale bars 200 µm are indicated ×10 objective, images are acquired in z-stack and here represented as maximum intensity projections.

Lymphocytes and hematopoietic cells require special culture media. Considering that in the MOAB bioreactor, all cells are perfused with the same culture medium, we needed to educate all the different cell types to grow in a common medium, namely, Stem Span™ SFEM. In order to construct a positive standard for tumor growth, we opted for a leukemic cell line (PLB-985). PLB-985 underwent conditioning to the common culture medium. The conditioning process comprised five steps, during which the proportions of RPMI/StemSpan™ SFEM media were gradually adjusted, transitioning from 100% RPMI/0% StemSpan™ SFEM to 75%/25% over 1 week, then to 50%/50% for another week, followed by 25%/75% for an additional week. Finally, the cells reached a stable cultivation state with 100% StemSpan™ SFEM. From this moment on, PLB-985 cells were successfully maintained in a stable StemSpan™ SFEM culture and exhibited robust growth. We tested the cells to assess their behavior in the new culture medium. We analyzed proliferation, viability, and apoptosis rates (Figures 3A–C). We referred to PLB-985 cells grown in RPMI as “normal” and those grown in StemSpan™ SFEM medium as “educated”. Importantly, none of the performed analyses showed any significant differences between the two strains. Proliferation assays, consisting of flow cytometry analysis, were conducted every 24 h for 4 days. As illustrated in Figure 3A, the proliferation rates were similar in normal and educated cells. Viability assays indicated a slightly reduced death rate in the educated condition (Figure 3B), albeit not significantly. Finally, apoptosis analysis revealed a similar percentage of live cells in both conditions, consistent with the previous assays, with mild variations in the proportions of early and late apoptotic and necrotic cells (Figure 3C). Therefore, we concluded that PLB-985 cells can be employed as a standard for leukemic growth in the StemSpan™ SFEM medium. Having established the viability of PLB-985 cells in the new medium, the next step was to test them in the bioreactor. Exploiting the optical accessibility of the MOAB, we manipulated the leukemic cells to introduce stable expression of green fluorescent protein (GFP), generating the GFP-PLB-985 substrain, for further experimentation.

Next, to determine the minimum number of cells to be present at time 0 and detectable after prolonged culture, we established a tumoral cell detection index (I_d_). Maintaining specified parameters (peristaltic pump rotation = 0.3 rpm, temperature = 37°C, and CO_2_ = 5%), we defined the tumoral detection index as the minimal absolute number of tumoral cells loaded into the MOAB system that allows detection after prolonged culture. For assessment, we seeded decreasing amounts of PLB-985-GFP^+^ leukemic cells (10,000, 1,000, 100, 10 cells) in fibronectin-functionalized silk fibroin scaffolds positioned in separate MOAB chambers. The system was then perfused and optically analyzed. Briefly, we successfully detected events after 2 weeks of dynamic culturing, down to the seeding condition corresponding to 100 PLB-985-GFP^+^ cells (Figure 3D). This I_d_ was considered in the design of subsequent experiments. In short, as little as 100 cells can lead to survival and tumor detection in the silk scaffold, a favorable value in comparison to around 5,000 cells required for soft agar colony assays (Borowicz et al., 2014).

### 3.4 The MOAB system is capable of identifying tumoral cells within a lymphoid niche at extremely low density, ensuring partial viability for up to 3 months

**FIGURE 4:**
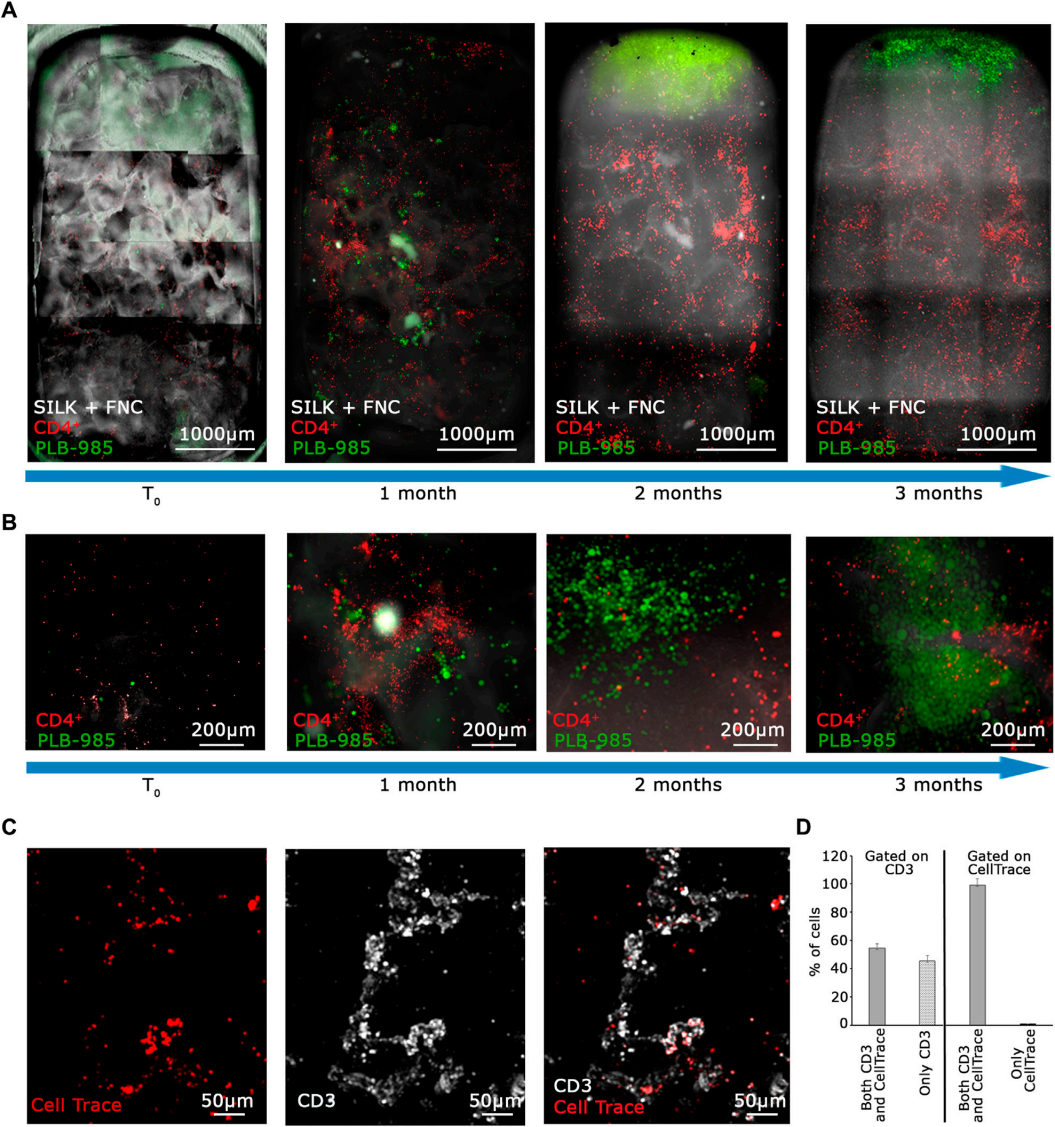
The MOAB System guarantees long-term viability of primary lymphocytes and expansion of tumor cells. **(A)** Evolution in time, as shown by blue arrows, of the lymphoid niche as visible from live imaging. Images represent the same sample at different time points. CD4^+^ T lymphocytes (CellTrace label, red LUT) and GFP^+^ leukemic cells (green LUT) seeded in the scaffold (white LUT). Images are mosaics of 15 tiled FOVs (×4 objective) acquired in z-stack and here represented as maximum intensity projections. **(B)** Higher objective magnification (×10) of representative areas of samples shown in **(A)**. **(C)** Higher magnification (×10 objective) acquisition of immunostaining post-fixation after 3 months of 3D dynamic culture in the bioreactor, showing colocalization of the CellTrace signal belonging to lymphocytes (red LUT) and CD3 membrane receptor (white LUT). **(D)** Quantification of 5 FOVs accounting for 3,298 total cells from images as represented in **(C)** shows relative percentages of CD3^+^ T cells positive for CellTrace reagent (left half of the bar-chart), and CellTrace^+^ cells positive for CD3 (right half). Scale bars are included.

As an initial step in validating the system as a tool for *in vitro* tumorigenicity research, we opted to assess its capability to provide optimal culturing conditions for the coculture of lymphocytes and PLB-985-GFP^+^ leukemic cells, all while enabling the detection of tumoral events at a long time. CD4^+^ T lymphocytes were labeled with Cell Trace Violet reagent to facilitate their optical detection in dynamic and mixed cultures. Strong proliferation weakens the signal by dilution, whereas its maintenance indicates homeostasis. We prepared a mixture of 100 leukemic PLB-985-GFP^+^ and 100,000 CD4^+^ T lymphocytes. All cells were seeded into the fibronectin-functionalized silk fibroin scaffold and cultivated for 3 days to enhance their adaptation to the scaffold. After this period, the silk was placed inside the bioreactor and perfused. We observed a clear scenario, as shown in Figures 4A, B. PLB-985 leukemic cells exhibited robust proliferation over the 3 months, as evident in Figure 4A (whole scaffold, green signal) and at higher magnification in Figure 4B (green signal). In addition, CD4^+^ T lymphocytes remained distinctly visible throughout the entire scaffold during the 3 months of dynamic culturing, and their signal maintained its intensity, suggesting minimal proliferation (Figures 4A, B, red signal).

To confirm the long-term viability of lymphocytes and assess the extent of proliferation in the MOAB, we conducted an immunofluorescence analysis on T lymphocytes after a prolonged co-culture. At the time of cell seeding, we labeled T cells with CellTrace Far Red (red signal in Figure 4C). Then, after 3 months, we fixed the scaffold and stained the seeded cells with an anti-CD3 antibody which labels the lymphocyte lineage (white signal in Figure 4C) by detecting the CD3 co-receptor exposed on the T lymphocyte plasma membrane. Live cells with intact membranes exhibit the CD3 protein. We scanned the entire scaffold and observed that almost all CellTrace^+^ T cells also exhibited a positive signal for CD3, as detailed in Figures 4C, D. Then, we gated our analysis on CD3^+^ T cells, and found that half of them were also positive for CellTrace. Taken together these data imply that a portion of the initial CD4^+^ T lymphocytes underwent intense proliferation, leading to a dilution of the CellTrace, whereas a part of CD4^+^ T lymphocytes are viable, not activated, and do not proliferate.

**FIGURE 5:**
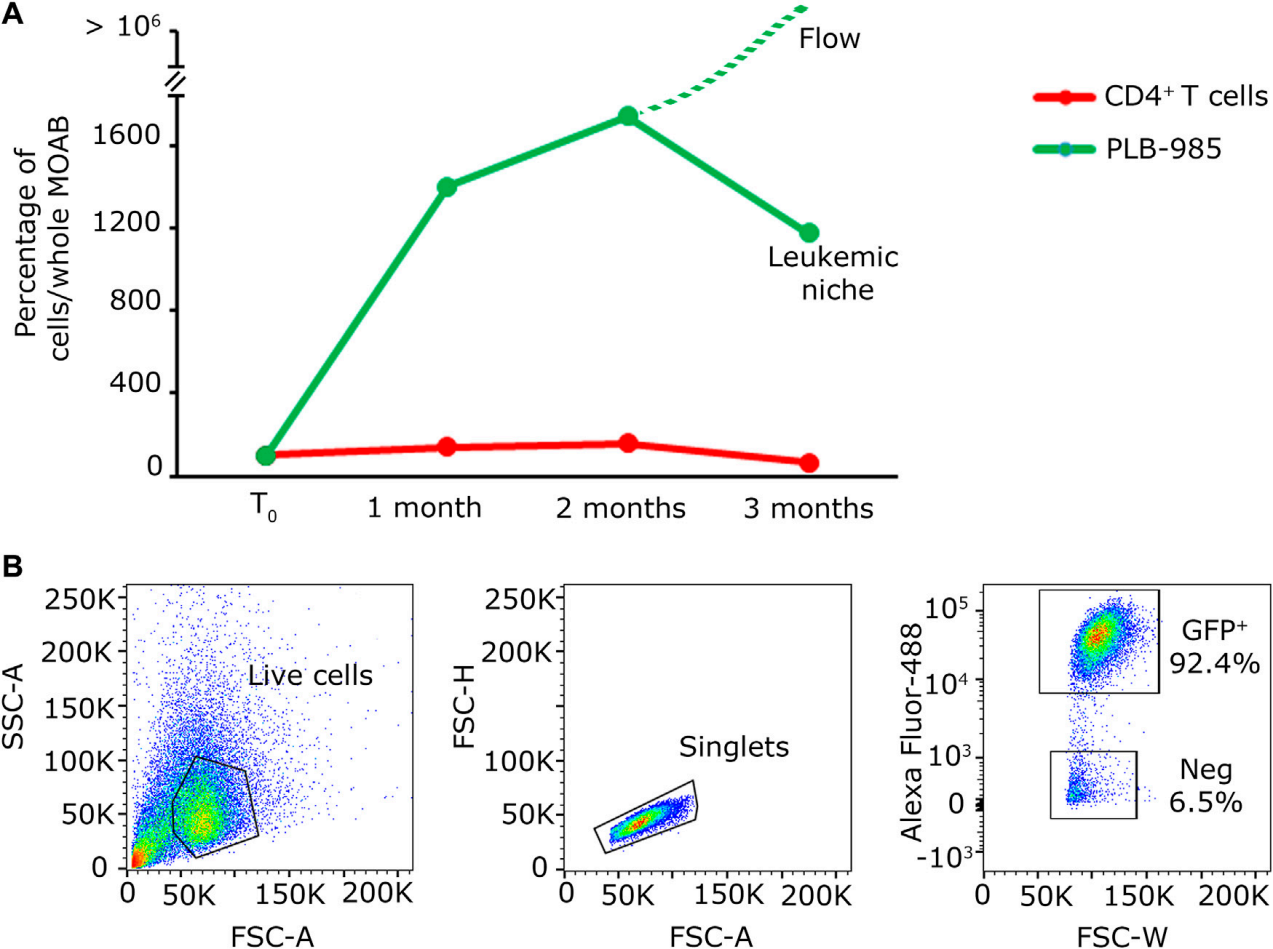
Quantification analysis of long-term co-culture of primary lymphocytes and leukemic cells. **(A)** Plot of the percentage of cells present within the whole scaffold, obtained from the images shown in Figure 4A. The red line identifies the evolution in time of CD4^+^ T cells; the green line represents the evolution in time of PLB-985 cells. The green dashed line indicates that the leukemic cells in culture tend to exit the scaffold after 2 months while maintaining their proliferative rate. **(B)** Cytofluorimetric analysis performed on the medium retrieved from the reservoir after the first 2 months of culture demonstrating the abundancy of leukemic GFP^+^ cells in the recirculating cells in the system.

The summarized results can be seen in the graph in Figure 4D, obtained by the analysis of randomly selected fields of view. Overall these data indicate that our system enables us to: a) visualize the massive expansion of tumor cells after 3 months of culture while b) maintaining a population of activated lymphocytes (CD3^+^/CellTrace^−^) mixed with lowly proliferating ones (CD3^+^/CellTrace^+^). Finally, we counted the number of cells of each cell population present within the scaffold. As shown in Figure 5A, the CD4^+^ T cells (red line) maintain the signal intensity over time, indicating minimal and constant proliferation. Moreover we examined sedimented cells in the reservoir and analyzed them by flow cytometry. As shown in Figure 5B cells in the reservoir are live and the higher percentage is positive for GFP, indicating that they are leukemic cells with a recirculation potential. Furthermore, we tested in CD4^+^ T cells the expression of CD25 as shown in Supplementary Figures S4A–C. With this analysis we could determine that already after 2 months of culture in the system, CD4^+^ lymphocytes show a higher level of activation with respect to the initial condition. In contrast, the leukemic cells (green line) show strong proliferation over time. Importantly, after 2 months of coculture, the PLB-985 cells, subjected to the constant flow of the MOAB, tend to leave the scaffold (flow), moving towards the reservoir, where they always maintain a high proliferative rate.

### 3.5 The MOAB is a promising platform for the detection of rare tumor events in the context of CD34^+^ hematopoietic precursors

**FIGURE 6:**
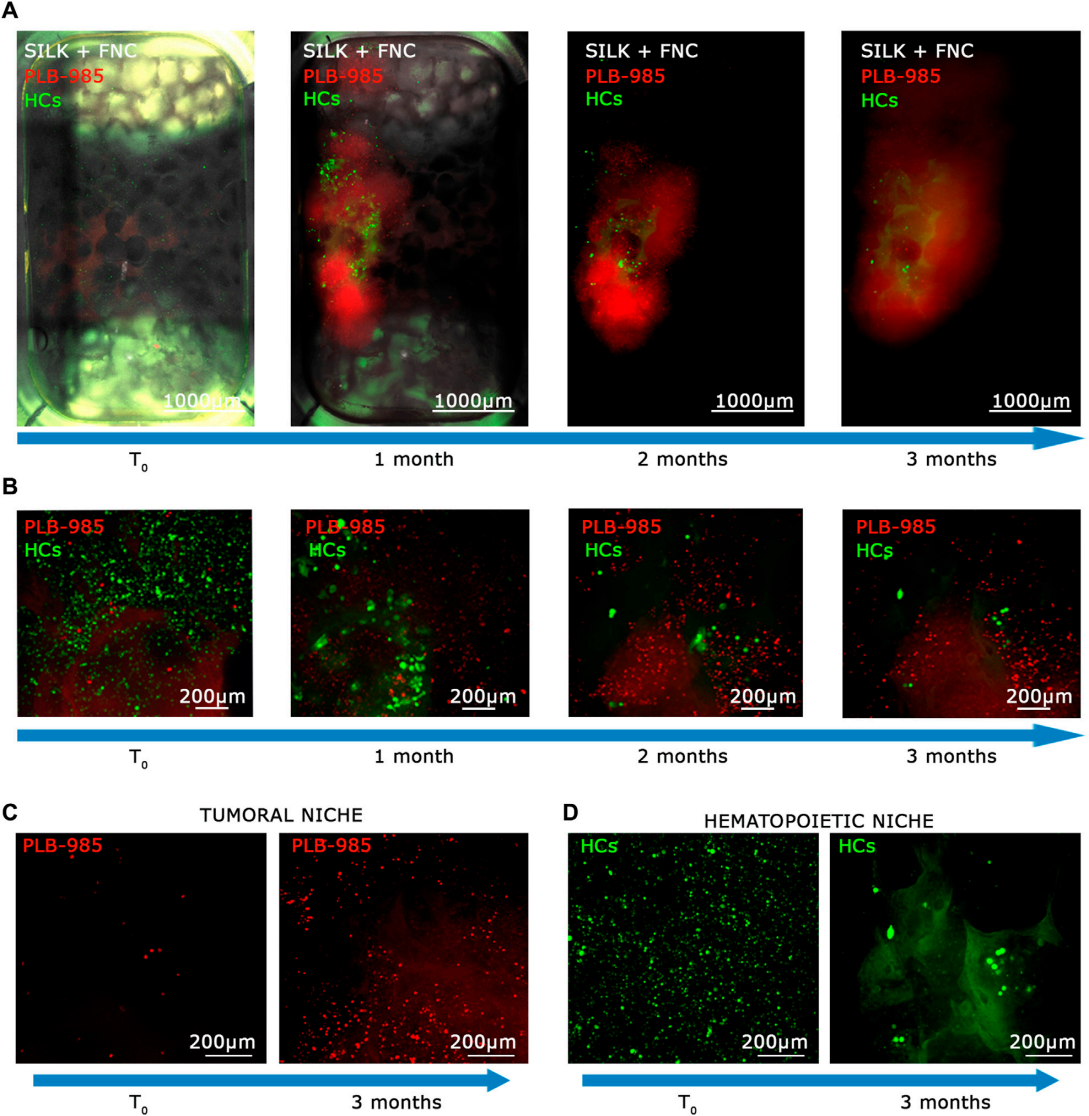
The MOAB System guarantees long-term viability of primary hematopoietic stem cells and expansion of tumor cells. **(A)** Evolution in time, as shown by blue arrows, of the hematopoietic niche as visible from live imaging. Images represent the same sample at different time points. GFP^+^ HCs (green LUT), and mCherry^+^ leukemic cells (red LUT), seeded in the scaffold (white LUT). Images are mosaics of 12 tiled FOVs (×4 objective) acquired in z-stack and here represented as maximum intensity projections. **(B)** Higher objective magnification (×10) of representative areas of samples shown in **(A)**. **(C, D)** Direct comparisons between final time point (3 months) and initial point visualized at higher objective magnification (×10) of representative areas, showing proliferating behavior of leukemic cells (**(C)**; red LUT) and showing the diminishing of HCs (**(D)**; green LUT). Scale bars are shown.

**FIGURE 7:**
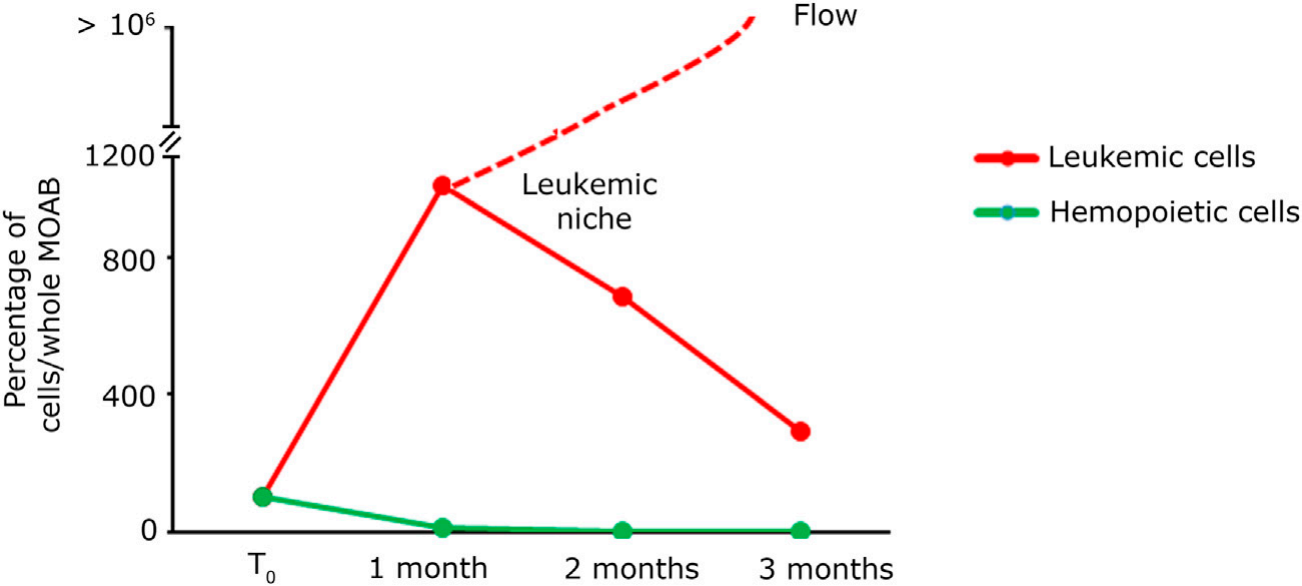
Quantification analysis of long-term co-culture of primary hematopoietic and leukemic cells. Plot of the percentage of cells present within the whole scaffold, obtained from the images shown in Figure 6A. The green line identifies the evolution in time of hematopoietic cells; the red line represents the evolution in time of leukemic cells. The red dashed line indicates that the leukemic cells in culture tend to exit the scaffold after the first month while maintaining their proliferative rate.

In light of the capability of our system to detect tumoral cells seeded at low density within a specific niche, and considering the potential of stem cell and gene therapy research, we decided to assess the bioreactor performance by the generation of a hematopoietic bone marrow-like niche. In this experiment, we mixed 100 PLB-985-mCherry^+^ leukemic cells with 100,000 CD34^+^-GFP^+^ human hematopoietic stem and progenitor cells (hHSPCs). CD34 staining is progressively lost, for this reason hHSPCs cells will be defined here in the subsequent time courses only as hemopoietic-derived cells (HCs). Similar to the lymphoid niche described in the previous section, all cells were initially seeded onto the fibronectin-functionalized silk fibroin scaffold. A static cultivation process lasting 3 days was employed to enhance their adaptation to the scaffold. Subsequently, the scaffold was introduced into the bioreactor and subjected to perfusion. Images representing the entire chamber and scaffold were examined to assess the presence and distribution of cells during long-term dynamic co-culture (Figures 6A, B). The co-culture of HCs and leukemic cells revealed within time a significant proliferation of the leukemic component (red signal in Figures 6A, B) and a noticeable reduction in the HCs (green signal in Figures 6A, B). Furthermore, the HCs, still visible after the first month, displayed a change in shape and dimension, consistently with the differentiation process (Figure 6B, green signal). After 3 months of dynamic coculturing, live HCs were reduced to very few, while leukemic cells greatly expanded and colonized the silk niche (Figures 6C, D). Taken together, the data suggest that our system can a) preserve the viability of HCs albeit not preventing their differentiation and b) simultaneously allow the continuous expansion of leukemic cells. Finally, we counted the number of cells of each cell population present within the scaffold. As shown in Figure 7, the hematopoietic cells (green line) reduce in number over time, coherently with differentiation and exhaustion. In contrast, the leukemic cells (red line) show strong proliferation over time. Importantly, already after the first month of coculture, the PLB-985 cells, subjected to the constant flow of the MOAB, tend to leave the scaffold (flow), moving towards the reservoir, where they always maintain a high proliferative rate.

## 4 Discussion

In this work, we describe a novel experimental approach that can be used for long-term culture of human primary CD4^+^ T lymphocytes and HCs. In addition, we provide proof of principle that our system can allow the clonal expansion and detection of rare tumorigenic cells.

First, we will delve into the biological significance of the cellular models that we have selected. Both CD4^+^ T lymphocytes and CD34^+^ hematopoietic stem and progenitor cells have their origin in the bone marrow (Mercier et al., 2012). The cultivation of these two types of primary cells is not only challenging but also holds relevance across multiple disciplines within medical biology (Prieto et al., 2021; Hiatt et al., 2022). CD4^+^ T cells are essential for adaptive immunity, their depletion, as seen in HIV-induced AIDS, leads to immunodeficiency, whereas their improper activation can trigger autoimmune disease (Geginat et al., 2013). For human T cells, including CD4^+^ T lymphocytes, the activation, typically through anti-CD3 and anti-CD28 stimulation, is generally required to proliferate and function effectively *in vitro* (Trickett and Kwan, 2003). Without such activation, T cells do not proliferate efficiently, and their inactive profile makes them harder to manipulate and profile. While the exact duration that human T cells can be kept *in vitro* without activation can vary, it is generally a challenge to sustain them for periods exceeding 4 weeks. Importantly, our long-term cultures of CD4^+^ T lymphocytes are performed without stimulation with anti-CD3 and anti-CD28.

Culturing human CD34^+^ cells *in vitro* presents a multitude of challenges. Among them, the main difficulty is the preservation of their stemness (Hatzmann et al., 2021). CD34^+^ cells are hematopoietic stem and progenitor cells and have the ability to differentiate into various types of blood cells (Kapoor et al., 2020). However, *in vitro* conditions can often induce spontaneous differentiation of these cells, thereby reducing their stemness and hence their utility for research and therapeutic applications. As such, establishing the optimal culture conditions to sustain the undifferentiated state of CD34^+^ cells, is a relevant challenge. This involves the careful balance of growth factors, cytokines, and other culture conditions. In our work, we used standard media for CD34^+^ cultivation, but given the millifluidic setup, cells could either remain grafted in the silk or enter circulation. However, although our studies suggest that the MOAB system does not adversely affect cell viability, morphological observation suggests that stemness is progressively lost. CD34^+^ cells’ functionality and differentiation status must be assessed in future studies. Nevertheless, the aim of this study was to detect clonogenic expansion of tumorigenic cells. Therefore, the loss of stemness was not an issue, but represents a potential advantage for detecting clonogenic expansion of proliferating cells.

In principle, seeding of CD34^+^ cells in the MOAB will lead to differentiation, unless clonogenic expansion of pre-tumorigenic cells occurs. Our findings demonstrate that the MOAB facilitates the expansion of tumorigenic cells in long-term cultures, thereby enabling their detection. Off-target editing is one of the main safety concerns for the use of genome editing in gene therapy (Zheng et al., 2020). These unwanted modifications can be positively selected *in vivo* and could lead to malignant transformation which renders tumorigenicity assessment of gene therapy products indispensable (Wang, 2023). In cell biology, the conventional *in vitro* assay for detecting tumorigenic events relies on colony formation in soft agar or, more broadly, on monitoring growth independent of anchorage. However, this assay has limited utility for HSPCs because a) HSPCs thrive under conditions of reduced attachment, and b) only highly malignant cells can overcome the selective pressure of growth in soft agar (Ozols et al., 1980). Another standard approach for assessing tumorigenicity involves xenotransplants, i.e., injecting cells into immunocompromised animals. While xenotransplants are extremely useful for testing *ex vivo* therapies on established tumors, they may, like the soft agar assay, require highly tumorigenic cells (Pierson et al., 2009). In the case of gene therapy of hematopoietic cells, malignancy may develop slowly with mutated pre-tumorigenic cells undergoing progressive clonal expansion and acquiring additional mutations (Stelmach and Trumpp, 2023). In other terms, in acute myeloid leukemia, rare clones may not be detectable at early stages but undergo extensive clonal competition over time, eventually resulting in a dominant population. In our setup, we provide proof of principle that the MOAB millifluidic system exerts a positive selection pressure toward a tumorigenic cell, allowing its expansion among normal cells. Thus, in principle, it could offer an efficient way to detect a tumorigenic cell through its clonal expansion in an environment that is not harshly selective and can also maintain non tumoral, normal cells. Additionally, clonogenic expansion can be followed by genomic analysis of integration events. For instance, sequencing or integration analysis of the starting edited population can be repeated up to 3 months after seeding cells, thus increasing the sensitivity of our analysis (Bulaklak and Gersbach, 2020). In short, our system partly mirrors the *in vivo* condition, with the primary difference being a relatively short timescale. In general, the cost and the difficulties of operating the MOAB system are limited. One of the advantages is that the system allows seeding of large number of cells, easily 100.000 per chamber, and many chambers can be run in parallel. To establish, however how many cells are required to detect a rare tumorigenic event is depending from the specific editing (Zhang et al., 2024). Future experiments can aim at defining with precision this number, perhaps defining an index that depends from the mutational burden.

Despite our millifluidic bioreactor system demonstrates significant potential for culture of primary lymphocytes and hematopoietic cells, it is important to acknowledge certain limitations inherent to its 3D structure. A key challenge lies in the difficulty of performing comprehensive cell viability assays and flow cytometry analyses on cells within the 3D scaffold. Retrieving live cells from the scaffold for molecular analyses is complex, making it difficult to perform analyses via flow cytometry. The process of cell recovery can be split into two sections: recovering circulating cells and recovering cells that have seeded in the silk. The recovery of circulating cells is straightforward: by changing the medium, you can recover the fraction of recirculated cells that are in the reservoir at that time, with a yield of over 50%. However, the retrieval of cells seeded for a long period in the MOAB is not efficient. Additionally, imaging techniques face obstacles due to light diffraction and autofluorescence from the scaffold, particularly affecting the visualization of weakly expressed markers or nuclear stains, like DAPI, at certain wavelengths. In summary, while *in situ* analysis is possible but with limits, methods for molecular and omics analyses such as single-cell RNA sequencing (sc-RNA seq) on target cells should be optimized. Another important issue concerns the sensitivity for testing true tumorigenic events. In our setup, we inoculated few tumor cells and waited weeks to see their clonal expansion. Ideally, the system should be employed for scoring unwanted tumorigenic events due to CD34^+^ manipulation. Our attempts to develop a context dependent positive control based on edited CD34^+^ cells were not successful due to oncogene-induced senescence and the difficulty to generate tumor cells from primary human cells, a far from simple issue which may require multiple hits and million of cells (Prieto et al., 2016). These limitations highlight areas for future improvement, including the development of non-invasive viability assays compatible with 3D structures, and the optimization of the positive control baseline.

**FIGURE 8:**
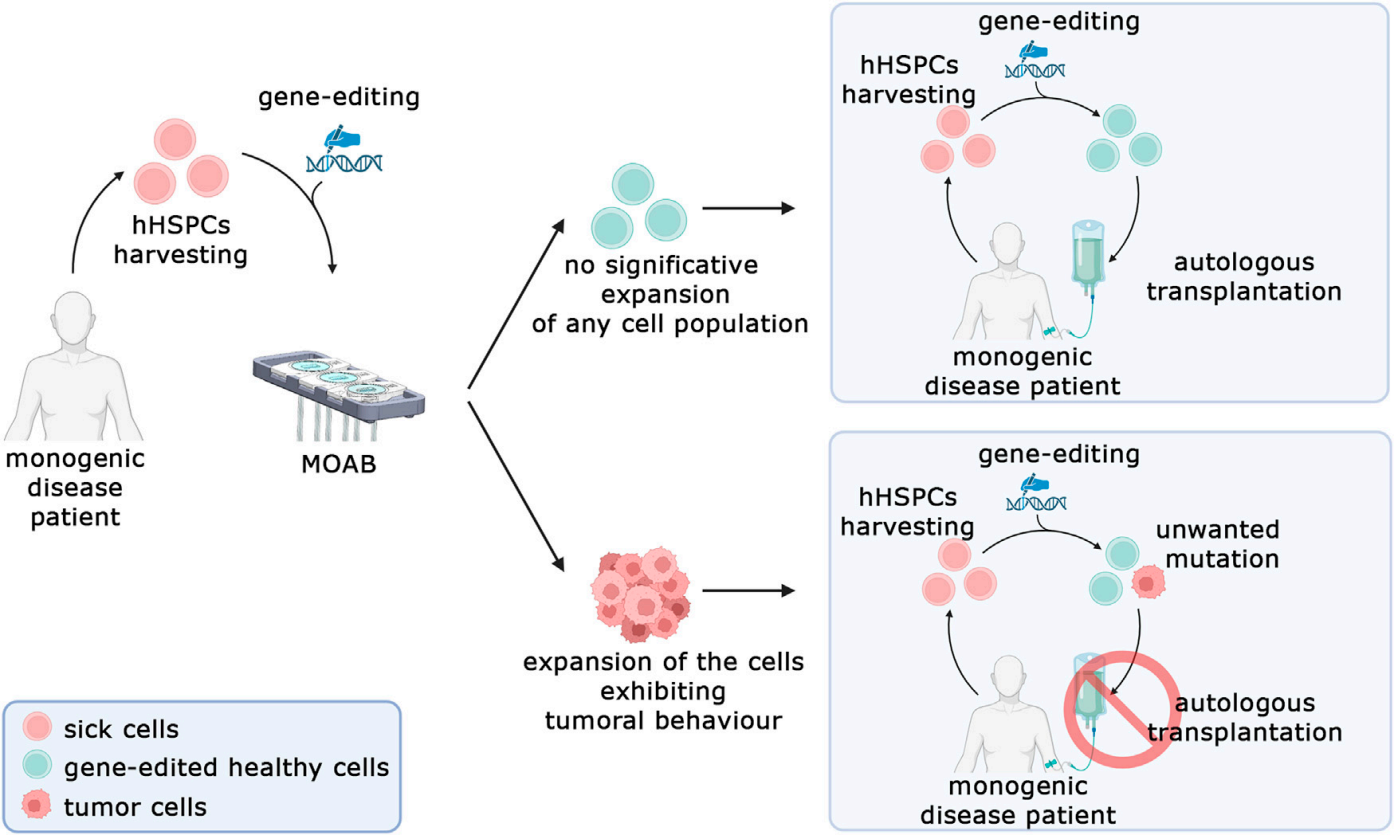
Prospective model for *in vitro* safety assessment of gene-edited human hematopoietic stem and progenitor cells (hHSPCs). The MOAB system aims to be used to test the expansion of cells bearing an unwanted protumorigenic mutation in gene-editing procedures. In case the test does not detect a significative expansion of cells, the autologous transplantation can be performed (top right), conversely if the test highlights a malignant phenotype, the cells cannot be employed for clinical applications and the autologous transplantation cannot be performed (Bottom, right). Created in BioRender.com.

Nevertheless, the system presented in this work represents a significant advancement in the detection of tumorigenic transformations in genome-edited stem cells. It offers a shift from traditional *in vivo* approaches to a safer, more reliable, and more ethical methodology. Our proposed solution is now primed for testing with a pool of primary cells that have undergone gene editing. Figure 8 illustrates the framework in which this system can be utilized. The process begins with the retrieval of hematopoietic stem cells from patients with monogenic diseases such as sickle cell anemia, thalassemia, or severe combined immunodeficiency. These cells undergo gene editing to correct the disease-causing aberrant gene. Subsequently, they can be tested in our system to investigate the potential expansion of malignant populations. If such an expansion is detected, it would indicate that the editing technique cannot be considered safe for clinical translation. Looking ahead, we envision several potential expansions of this system. One promising avenue is the addition of a second model housed in another chamber of the bioreactor, recreating a homing environment for transformed cells.

In conclusion, we have adapted a new millifluidic model, based on the MOAB network that enables cultivation of primary human cells, specifically CD4^+^ T lymphocytes and HCs. Our system provides a compelling proof-of-concept, demonstrating its efficiency in identifying rare tumorigenic cells in circulation. We can detect as little as 100 seeded cells, which is significantly less than soft agar systems. With further refinement, this system holds the potential to serve as a cost-effective, sensitive alternative for detecting tumorigenic occurrences post gene therapy-based editing.

## Data Availability

The raw data supporting the conclusions of this article will be made available by the authors, without undue reservation.
