# Supplementary figures and images for: A millifluidic bioreactor allows the long term culture of primary lymphocytes or CD34+ hematopoietic cells while allowing the detection of tumorigenic expansion
Source: Front Bioeng Biotechnol. 2024 Oct 2;12:1388312. doi: 10.3389/fbioe.2024.1388312 (PMC11479935; doi:10.3389/fbioe.2024.1388312)

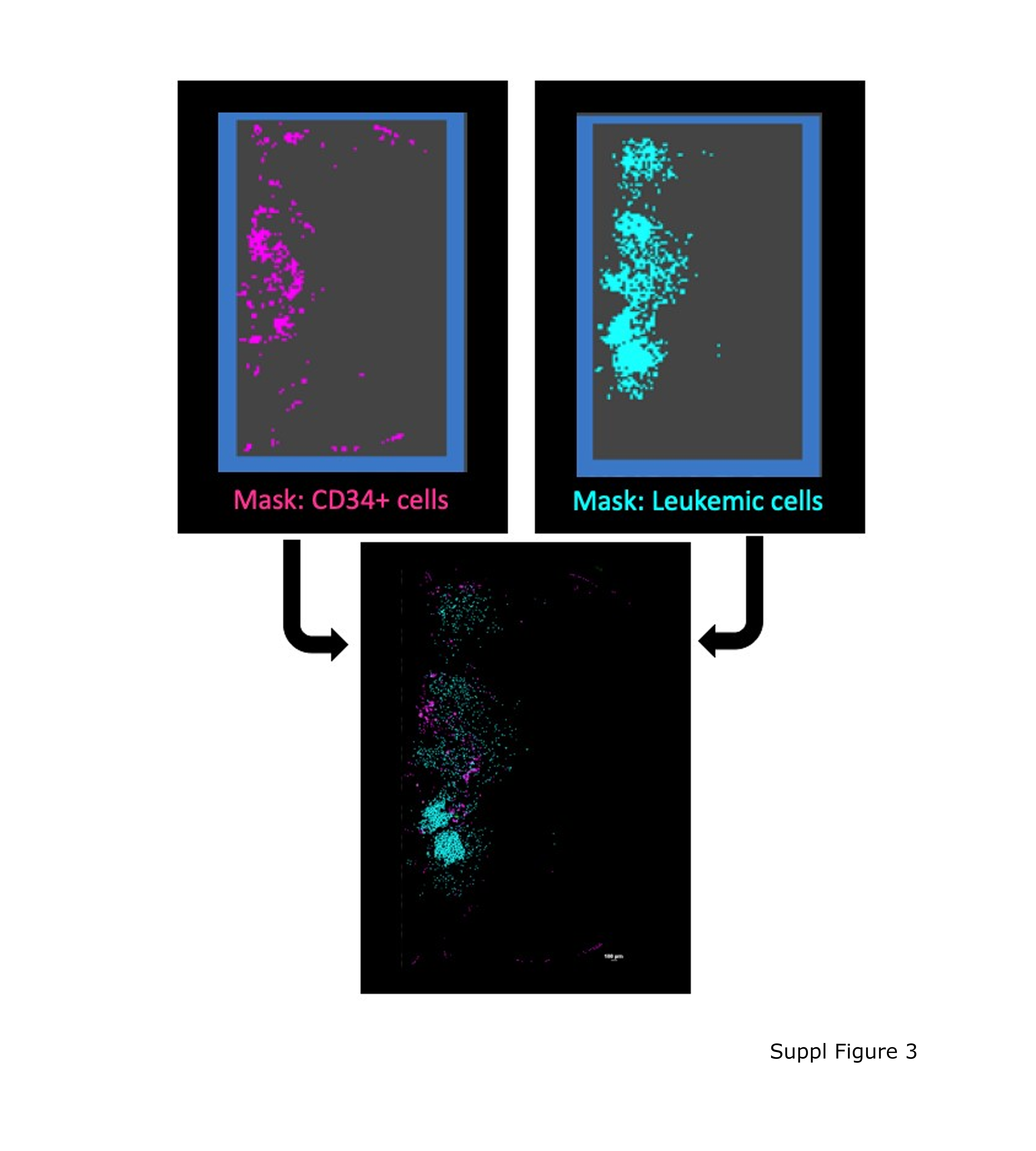

Supplement: Supplementary file 1 [file Image3.TIF]

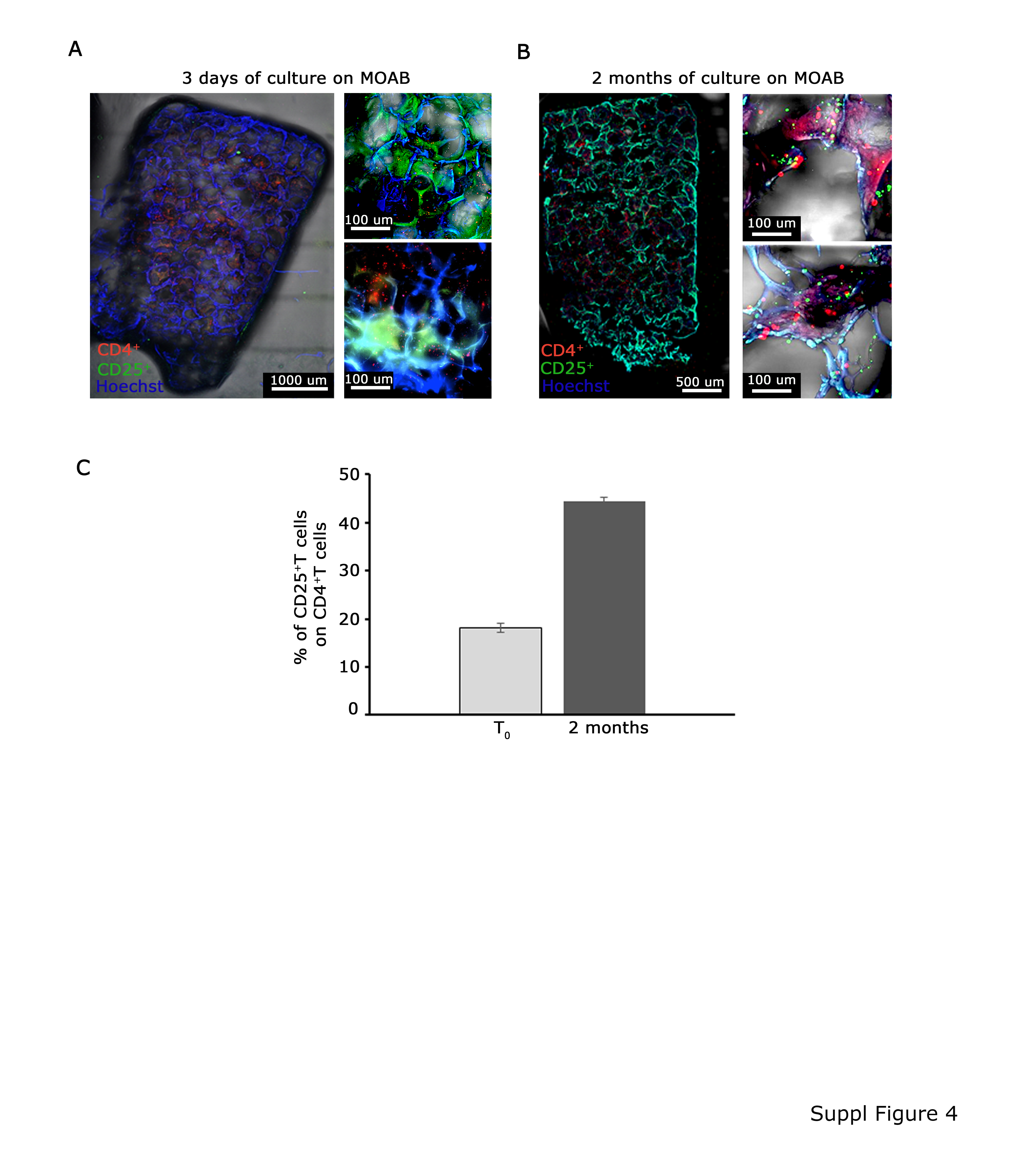

Supplement: Supplementary file 2 [file Image4.TIF]

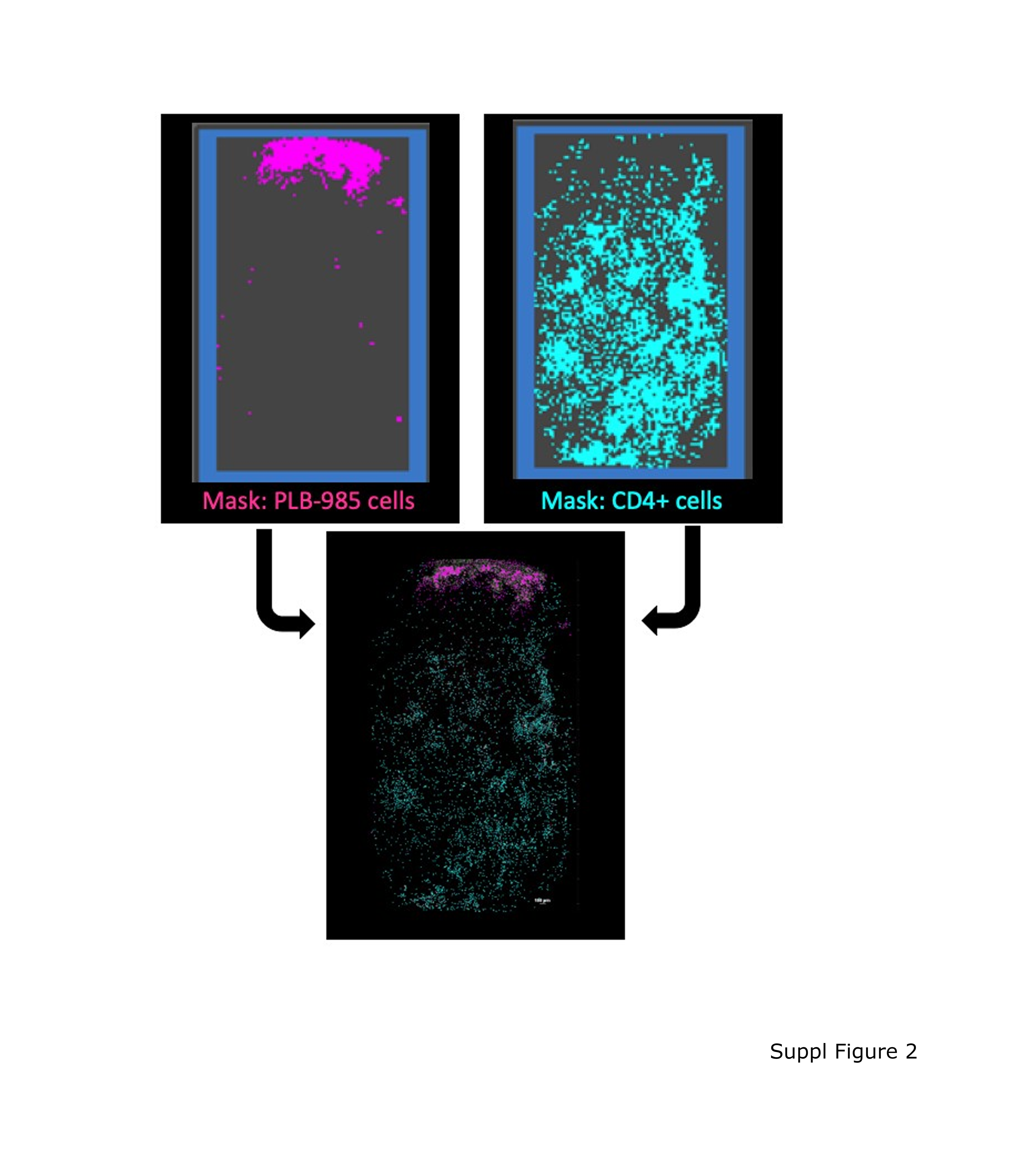

Supplement: Supplementary file 3 [file Image2.TIF]

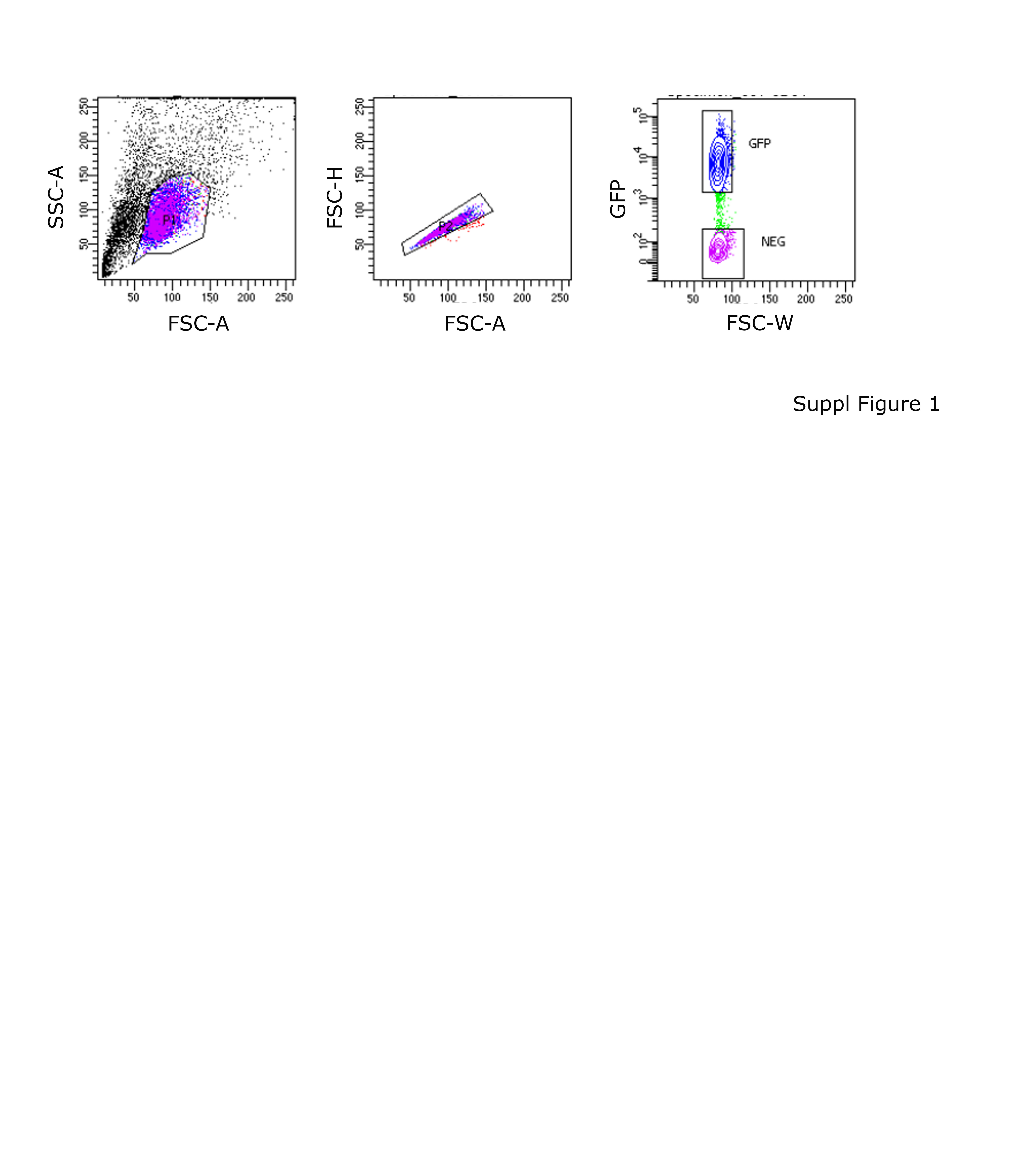

Supplement: Supplementary file 4 [file Image1.TIF]
